# Platelet-to-lymphocyte ratio and the first occurrence of peritonitis in peritoneal dialysis patients

**DOI:** 10.1186/s12882-022-03038-5

**Published:** 2022-12-30

**Authors:** Ning Su, Yijia Zheng, Xing Zhang, Xingming Tang, Li-wen Tang, Qinqin Wang, Xingyu Chen, Xiaoyang Wang, Yueqiang Wen, Xiaoran Feng, Qian Zhou, Jiafan Zhou, Yafang Li, Sijia Shang

**Affiliations:** 1 Department of Nephrology, The Sixth Affiliated Hospital, Sun Yat-Sen University, Guangzhou, China; 2 Department of Nephrology, DongGuan SongShan Lake Hospital, Dongguan, China; 3 Department of Nephrology, The First Affiliated Hospital of Zhengzhou University, Zhengzhou, China; 4 Department of Nephrology, The Second Affiliated Hospital, Guangzhou Medical University, Guangzhou, China; 5 Department of Nephrology, Jiujiang No. 1 People’s Hospital, Jiujiang, China; 6 Department of Medical Statistics, Clinical Trials Unit, The First Affiliated Hospital, Sun Yat-Sen University, Guangzhou, China; 7 Department of Hematology, The Sixth Affiliated Hospital, Sun Yat-Sen University, Guangzhou, China; 8 Department of Intensive Care Unit, The Sixth Affiliated Hospital, Sun Yat-Sen University, Guangzhou, China

**Keywords:** Platelet-to-lymphocyte ratio, Peritoneal dialysis, Peritonitis

## Abstract

**Background:**

Platelet-to-lymphocyte ratio (PLR) has been used as a potential biomarker of inflammation-related diseases, but its role in the peritoneal dialysis-related peritonitis (PDRP) is still uncertain. This study was aimed to investigate the association between PLR and the new-onset PDRP in peritoneal dialysis (PD) patients.

**Methods:**

In this multicenter retrospective study, 1378 PD Chinese PD patients were recruited from four centers, who were divided into the high PLR group (HPG) and the low PLR group (LPG) according to the cutoff value of PLR. The correlation between PLR and the new-onset PDRP was assessed using the Cox regression model analysis.

**Results:**

During follow-up, 121 new-onset PDRP events were recorded. Kaplan–Meier survival curve showed a higher risk of new-onset PDRP in the HPG (log-rank test, *P* < 0.001). After adjusting for confounding factors, the Cox regression model showed the risk of new-onset PDRP was higher in the HPG than that in the LPG (HR 1.689, 95%CI 1.096–2.602, *P* = 0.017). Competitive risk model analysis showed that significant differences still existed between the two PLR groups in the presence of other competitive events (*P* < 0.001).

**Conclusion:**

PLR is independently associated with the new-onset PDRP in PD patients.

**Supplementary Information:**

The online version contains supplementary material available at 10.1186/s12882-022-03038-5.

## Introduction

Peritoneal dialysis (PD) is a primary treatment for end-stage renal disease (ESRD) [1]. Peritoneal dialysis-related peritonitis (PDRP) is a common and serious complication of PD patients [2]. According to the International Society for Peritoneal Dialysis (ISPD), the peritonitis rate should not be higher than 0.5 cases per patient-year [3]. However, the incidence of PDRP reported by different countries and different centers within the same country varies greatly [4]. According to the Peritoneal Dialysis Outcomes and Practice Patterns Study (PDOPPS), more than 0.50 per patient-year was reported in 10% of facilities [5]. PDRP is the leading cause of PD failure, which results in considerable morbidity, mortality, and health care costs [4, 6–8]. The occurrence of PDRP is related to chronic inflammation. The continuous expression of inflammation stimulates the intestinal tract, which may lead to intestinal dysfunction, bacterial translocation and infection [9]. Studies have shown that inflammatory markers IL-6 and CRP may be potential predictors of peritonitis in PD patients [10]. However, the cost of CRP and IL6 test is relatively expensive, thus limiting the application among some patients. To improve the clinical outcome of PD, there is a need for facile and affordable biomarkers to identify patients at risk of PDRP and to guide personalized interventions.

Platelet-to-lymphocyte ratio (PLR) is an inexpensive, replicable, and easily measurable hematological index. It is originally developed as a predictor of tumor prognosis and is associated with inflammation in cancer patients [11, 12]. Increased platelet counts and decreased lymphocyte counts have been shown to be related to both aggregation and inflammation [13]. Recent studies have found that higher PLR is associated with the inflammatory state and poor prognosis of 2019-novel coronavirus disease and sepsis [14, 15]. In dialysis and non-dialysis patients with chronic kidney disease(CKD), PLR has been reported to be associated with inflammation [16]. And some studies also have shown that PLR is associated with all-cause mortality and the prognosis of cardiovascular events in patients with CKD [16–19]. To date, no studies have revealed the association between PLR and PDRP in PD patients.

The purpose of the present study was to assess whether PLR is correlated with new-onset PDRP in PD patients.

## Methods

### Patients

In this multicenter retrospective study, a total of 1378 patients from four peritoneal dialysis centers in China were recruited from January 31, 2003, to February 21, 2020. All patients received continuous ambulatory peritoneal dialysis (CAPD). Platelet and lymphocyte counts were available in these patients. Of them, 127 were excluded for the following reasons: age younger than 18 years or older than 80 years (*n* = 36), PD was maintained for less than 3 months (*n* = 42), PD was maintained for more than 10 years(*n* = 10), clinical evidence of active infection (*n* = 14), history of hematological or autoimmune disease and taking glucocorticoid or immunosuppressive (*n* = 25). The above patients were excluded because those factors may influence the PLR level. This study was approved by the Ethics Committee of the Sixth Affiliated Hospital of Sun Yet-Sen University (No. 2021SLYEC-177). Informed consent was obtained from all subjects and their legal guardians.

The study was performed in accordance with the principles of the Declaration of Helsinki.

### Baseline investigations

Baseline demographic and clinical data were collected at the initiation of PD therapy. Laboratory indicators were collected one week before the first PD treatment in most patients, and in order to ensure the integrity of the data, the data collection time was extended to 30 days after the start of PD treatment. The same laboratory inspection machinery was used in each center. Routine blood tests were performed using automatic hematology analyzer (SysmexXN-2000 or MindrayCAL6800). Absolute blood platelet counts were divided by absolute blood lymphocyte counts to obtain the PLR. The diagnosis of PDRP was made if the patient had at least two of the following criteria according to the 2017 Internation Society for Peritoneal Dialysis (ISPD) guidelines: 1) abdominal pain with or without cloudy peritoneal dialysis effluent, with or without fever; 2) total leukocyte count ≥ 100 × 10^6^ cells/L, with more than 50% polymorphonuclear cells in the differential count; and 3) positive Gram staining or culture of peritoneal dialysis effluent [20].

The diagnostic criteria of diabetes are based on Standards of Medical Care in Diabetes [21]. Hypertension was recorded if the patient took antihypertensive drugs or had two separate blood pressure measurements ≥ 140/90 mmHg. Patients were returned quarterly to their respective centers for evaluation and were interviewed by trained nurses over the phone to assess their general condition each month [22].

### Study outcome

The outcome was the first occurrence of PDRP since PD therapy. The endpoint of follow-up was the new-onset PDRP, death, transfer to hemodialysis therapy, renal transplantation, transfer to other centers, or censoring on April 01, 2020. Peritoneal dialysis patients were followed up by trained graduate students and nurses.

### Statistical analysis

Kaplan–Meier curves and log-rank test were used to examine the difference in the cumulative hazard among the two groups. Based on the receiver operating characteristic (ROC) analysis results, according to the Youden index, the optimal cutoff value of PLR was 161.5.

Through normality test, only age and body mass index (BMI) conformed to normal distribution. Age and BMI were expressed by mean ± standard deviation. And other skewness distribution indexes were expressed by median (interquartile range). Categorical variables were compared with the Pearson χ2 test, and the results are presented as frequencies (percentages). Differences between the PLR groups were tested using Kruskal–Wallis test for skewed continuous variables and an independent sample t-test was used for continuous variables.

The univariable cox regression model was used to examine the association between patients’ characteristics and new-onset PDRP. The factors included in the Cox regression model were determined according to the results of univariate Cox regression analysis and previous studies. Three Cox proportional hazards regression models were conducted to examine the association between PLR and the first occurrence of PDRP: model 1, demographic; model 2, model 1 plus comorbid conditions; model 3, model 2 plus laboratory variables.

The interaction between the subgroup variables (age, sex, hyperlipemia, diabetes) and the PLR group was tested by performing a formal test of interaction. Forest plots were used to show the relationship between PLR and new-onset PDRP in each subgroup. Competitive risk models were used to investigate the effects of death, transfer to hemodialysis, and transfer to kidney transplantation events.

The statistical analysis was done by SPSS 25.0, R software (version R 4.1.0 www.r-project.org), and GraphPad Prism8. All the tests were carried out bilaterally, and all the tests with *P* < 0.05 were considered to be meaningful.

## Results

### Patient characteristics

**Fig. 1:**
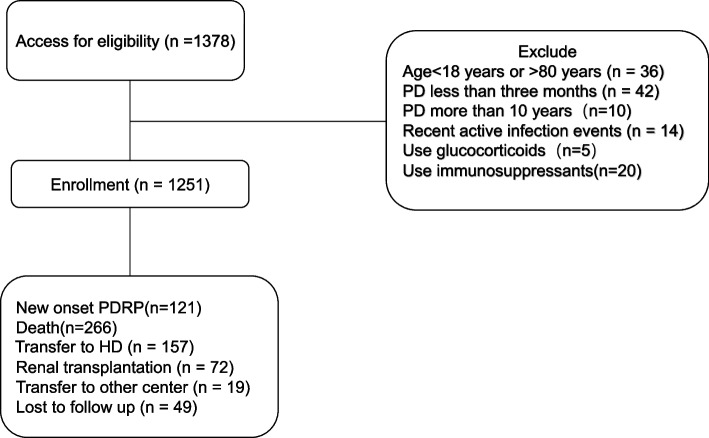
The flow chart shows the exclusion and selection of patients

**Fig. 2:**
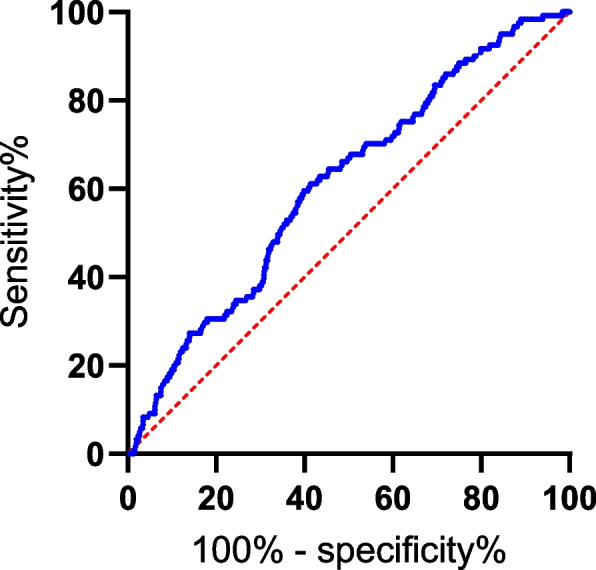
ROC curves analysis for PLR. The AUC of the PLR (0.611,95% CI: 0.560–0.662, *P *< 0.001)

Finally, a total of 1251 patients were included in the statistical analysis. At a mean follow-up of 45.45 months, 121 patients experienced the first occurrence of PDRP (Fig. 1). In order to obtain the optimal cutoff value of PLR, we analyzed the ROC curve with peritonitis as the state variable (Fig. 2). The optimal cutoff value of PLR is 161.5.

There were 712 patients in PLR < 161.5 group (low PLR group, LPG), and 539 patients in PLR ≥ 161.5 group (high PLR group, HPG). In the total patients, the age was 50.68 ± 14.58 years, and 719(57.5%) of patients were male. There were 939 (75.1%) patients with hypertension, 299 (23.9%) patients with diabetes, 78 (6.2%) patients with hyperlipidemia, and 114 (9.1%) patients with a history of cardiovascular disease. The patients in HPG were older, with higher rates of diabetes and a history of hyperlipidemia and CVD. Other laboratory indicators were listed in Table 1.

**Table 1:** Demographic and laboratory values of 1251 PD patients

	Total	PLR < 161.5	PLR ≥ 161.5	*P*-value
Numbers	1251	712	539	-
**Demographics**				
Age(years)	50.68 ± 14.58	49.18 ± 14.77	52.66 ± 14.1	< 0.001
Male(%)	719(57.5%)	398(55.9%)	314(59.6%)	0.189
Smoke (%)	63(5.0%)	29(4.1%)	34(6.3%)	0.073
BMI (kg/ m^2^)	22.10 ± 3.38	22.13 ± 3.32	22.06 ± 3.47	0.735
**Comorbidities**				
Hypertension (%)	939(75.1%)	528(74.2%)	411(76.3%)	0.396
Diabetes (%)	299(23.9%)	150(21.1%)	149(27.6%)	0.007
History of hyperlipemia (%)	78(6.2%)	35(4.9%)	43(8.0%)	0.027
History of CVD (%)	114(9.1%)	53(7.4%)	61(11.3%)	0.018
**Laboratory Variables**				
Total Kt/V	2.20(1.71–2.72)	2.20(1.72–2.69)	2.19(1.70–2.76)	0.559
Albumin (g/L)	35.30(31.50–38.50)	35.90(32.30–39.00)	34.80(30.60–38.00)	0.003
RRF(mL/min/1.73 m^2^)	3.22(1.68–5.65)	3.11(1.64–5.60)	3.37(1.79–5.71)	0.313
WBC (× 10^9^/L)	5.90(4.63–7.31)	5.83(4.66–7.20)	5.97(4.60–7.47)	0.610
RBC (× 10^12^/L)	2.86(2.45–3.35)	2.78(2.39–3.31)	2.87(2.45–3.39)	0.058
Hemoglobin(g/L)	82.00(71.00–97.00)	80.00(70.00–95.75)	84.00 (70.00–98.00)	0.085
Lymphocyte (× 10^9^/L)	1.17(0.88–1.48)	1.31(1.04–1.64)	0.95(0.73–1.2.0)	< 0.001
Neutrophil (× 10^9^/L)	3.92(3.00–5.10)	38.00(2.91–4.96)	4.29(3.14–5.6)	< 0.001
Platelet (× 10^9^/L)	174.00(130.00–226.25)	149.50(105.00–188.00)	214.00(169.00–266.00)	< 0.001
PLR	147.76(106.86–200.65)	113.52(90.88–136.73)	214.08(184.21–267.01)	< 0.001
FBG (mmol/L)	4.70(4.18–5.55)	4.67(4.2–5.34)	4.9(4.2–5.8)	< 0.001
Urea nitrogen(mmol/L)	20.42(15.6–26.93)	21 (16.1–27.3)	19.8(15.1–26.4)	0.041
Calcium (mmol/L)	2.04(1.87–2.19)	2.03(1.85–2.19)	2.06(1.89–2.21)	0.032
Phosphorus (mmol/L)	1.72(1.38–2.06)	1.72(1.39–2.07)	1.70(1.35–2.02)	0. 551
iPTH (pg/ml)	179.00(87.60–290.75)	192.40(100.51–310.66)	158.98(60.20–264.10)	0.005
Total cholesterol(mmol/L)	4.21(3.45–5.77)	4.11(3.42–4.97)	4.31(3.53–5.02)	0.018
Triacylglycerol(mmol/L)	1.32(0.93–1.82)	1.27(0.93–1.81)	1.33(0.95–1.86)	0.206

*BMI* body mass index, *CVD* cardiovascular disease, *RRF* residual renal function, *WBC* white blood cell, *RBC* red blood cell, *PLR* platelet lymphocyte ratio, *FBG* fasting blood glucose

### PLR and PDRP

Variables associated with new-onset PDRP were analyzed with univariable and multivariable Cox regression analysis. History of smoking, CVD, and the labs including serum albumin, serum calcium, serum potassium, and hemoglobin, were associated with new-onset PDRP (Table2).

**Table 2:** Significant risk factors for the first occurrence of PDRP

Variables	HR	95%CI	*P*-value
**Univariable Cox regression**			
BMI (per 1-kg/m2 greater)	1.096	1.042–1.152	< 0.001
Smoke history (yes vs. no)	7.532	4.895–11.589	< 0.001
CVD history (yes vs. no)	4.755	3.191–7.084	< 0.001
Hyperlipemia history (yes vs. no)	8.232	5.577–12.152	< 0.001
Diabetes (yes vs. no)	2.588	1.792–3.737	< 0.001
WBC (per 10^9^/L greater)	1.100	1.027–1.178	0.006
Hemoglobin (per 1-g/L greater)	1.038	1.032–1.045	< 0.001
Serum albumin (per 1-g/L greater)	0.934	0.905–0.964	< 0.001
Serum calcium (per 1-mmol/L greater)	9.130	4.944–16.859	< 0.001
Serum phosphorus (per 1-mmol/L greater)	0.380	0.255–0.565	< 0.001
Serum potassium (per 1-mmol/L greater)	0.399	0.320–0.498	< 0.001
**Multivariable Cox regression**			
Smoke history (yes vs. no)	2.954	1.847–4.724	< 0.001
CVD history (yes vs. no)	1.770	1.114–2.811	0.016
Hemoglobin (per 1-g/L greater)	1.021	1.012–1.031	< 0.001
Serum albumin (per 1-g/L greater)	0.924	0.891–0.959	< 0.001
Serum calcium (per 1-mmol/L greater)	3.579	1.527–8.387	0.003
Serum potassium (per 1-mmol/L greater)	0.626	0.483–0.812	< 0.001

*PDRP* peritoneal dialysis-related peritonitis, *BMI* body mass index, *CVD* cardiovascular disease, *WBC* white blood cell, *HR* hazard ratio, *CI* confidence interval

With the LPG as a reference, after adjusting for demographic indicators, medical history, and laboratory indicators, Cox multivariable analysis showed that the high PLR group had a 2.235(95% CI 1.539–3.245, *P* < 0.001), 1.711(95% CI 1.176–2.491, *P* = 0.005) and 1.689(95% CI 1.096–2.602, *P* = 0.017) times higher risk of new-onset PDRP (Table 3).

**Table 3:** Relationship Between PLR and the new-onset PDRP

Risk factor	HR (95% CI)	*P*-value
Unadjusted	2.268(1.573–3.271)	< 0.001
Model1	2.235(1.539–3.245)	< 0.001
Model2	1.711(1.176–2.491)	0.005
Model3	1.689(1.096–2.602)	0.017

Model 1: age, BMI, sex

Model 2: Model 1 plus smoking history, hyperlipemia, diabetes, cardiovascular disease

Model 3: Model 2 plus WBC, RBC, serum albumin, serum creatinine, serum uric acid, FBG, total cholesterol, serum phosphorus, serum alkaline phosphatase, iPTH, serum calcium, serum potassium

*WBC* white blood cell, *RBC* red blood cell, *BMI* body mass index, *FBG* fasting blood glucose, *iPTH* intact parathyroid hormone, *HR* hazard ratio, *CI* confidence interval

According to Fig. 2, the area under the ROC curve of the PLR (0.611, 95% CI: 0.560–0.662, *P* < 0.001). The sensitivity and specificity of PLR were 61.2% and 58.8% respectively.

**Fig. 3:**
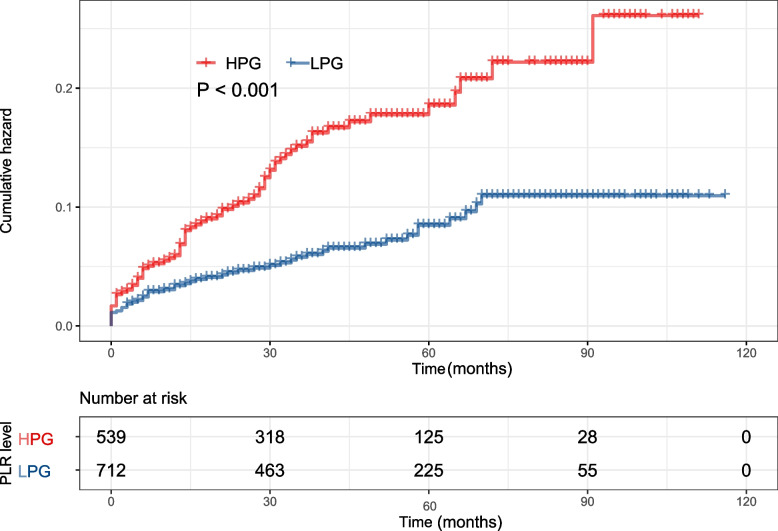
Kaplan–Meier survival curve for PLR on new onset PDRP event

**Fig. 4:**
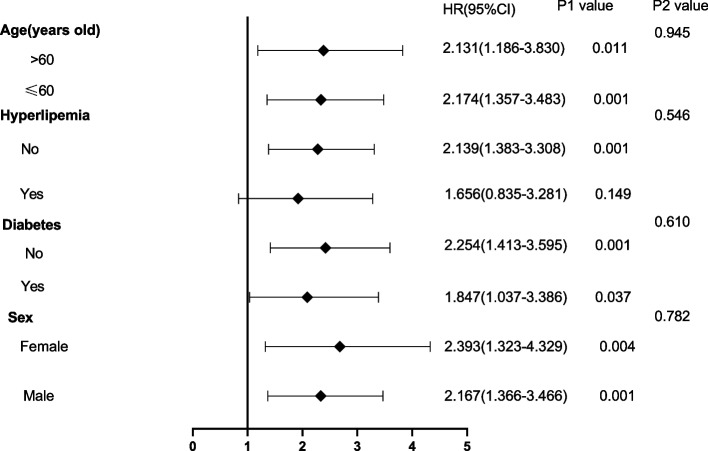
Forest plot of relationship between PLR and thenew onset PDRP in different subgroups. The P1value corresponded to the relationship between PLR and the new onset PDRP in different subgroups. The P2value corresponded to the interaction test between the PLR and the subgroups variable of interest

The Kaplan–Meier cumulative incidence curve demonstrated that the patients in HPG had a higher incidence of new-onset PDRP (log-rank test, *P* < 0.001) (Fig. 3) than the patients in LPG. The forest plot showed that there was no interaction between age, sex, hyperlipemia, diabetes, and PLR (Fig. 4). The relationship between PLR and PDRP has a similar pattern among subgroups.

**Fig. 5:**
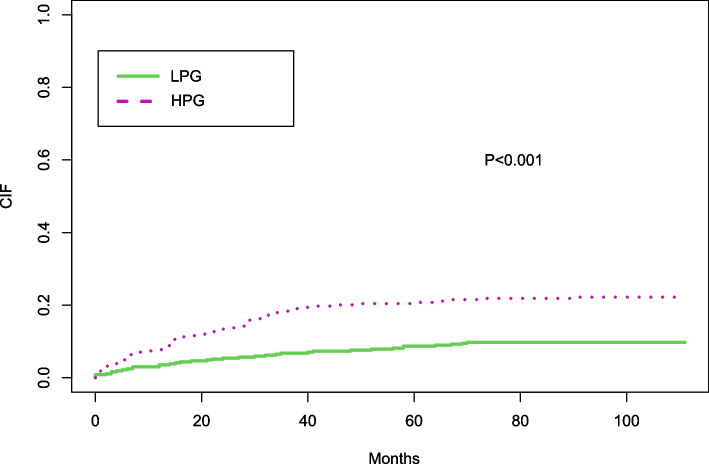
Competitive risk models for PDRP outcomes and other competitive events. Estimates of cumulative incidence curves between new onset PDRP events and other competing events at different PLR levels. The cumulative incidence of new onset PDRP event was significantly different between the two groups (*P* < 0.001)

In the competitive risk model, the cumulative incidence function (CIF) of new-onset peritoneal-associated peritonitis was significantly different between different PLR groups after considering the influence of competitive risk events such as death, kidney transplantation, and hemodialysis (*P *< 0.001) (Fig. 5).

## Discussion

This is a multicenter retrospective study to investigate the relationship between PLR and the development of new-onset PDRP in PD patients, suggesting that a higher PLR lever was associated with a higher risk of PDRP.

Chronic systemic inflammation is an important adverse effect in PD patients. This may be related to the accumulation of uremic wastes, PD catheterization, bioincompatible dialysate, and periodontal problems [23]. It is well known that platelets and lymphocytes come from the same hematopoietic stem cell [24], and PLR should be maintained in constant homeostasis. Higher PLR conditions mean relatively high platelets and/or low lymphocytes. Many studies have proved that PLR is considered as an indicator of systemic inflammatory response when patients have no obvious infection [25]. Higher platelet count may reflect the increase of platelet activation [26]. Both platelets and lymphocytes were closely associated with infection in patients with ESRD. Activated platelets secrete a large number of inflammatory mediators and chemokines such as vasoactive amine, interleukin-1, and proteolytic enzyme, which directly or indirectly cause target cell activation and trigger an inflammatory reaction [24, 26]. At the same time, platelets play a significant role in leukocyte recruitment, activation, and migration [27]. The expression of platelet P-selectin and the subsequent formation of platelet-leukocyte aggregates up-regulate the pro-inflammatory function of leukocytes. Activated platelets can stimulate leukocytes to recruit to the blood vessel wall and cause inflammation [28]. Lymphocytopenia reveals the inhibition of congenital cellular immunity, which may be caused by systemic inflammation and may lead to inadequate immune response and weakened defense [29]. When platelets and lymphocytes are considered together, elevated PLR may indicate poor physical condition and chronic inflammation in patients with chronic kidney disease.

Different cut-off values for PLR have been used in clinical studies. This depends on the complications, such as cancer, hematology, sepsis, cardiovascular disease, diabetes, and the type of endpoint such as morbidity and/or prevalence of the disease. Elevated PLR value can predict a variety of diseases. A study by Stefan Diem showed that high baseline PLR was significantly associated with poorer overall survival (OS) in patients with NSCLC [11]. Wang reported an association between elevated PLR and poor OS (HR = 1.85,95% CI 1.51–2.25, *P* < 0.001) in prostate cancer patients [30]. Elevated PLR indicates systemic inflammation, which can lead to increased resting energy expenditure, hypoproteinemia, and malnutrition, ultimately leading to weight loss and tumor progression, leading to increased mortality [12]. Yun Suk G. et al. 's study showed that elevated PLR was associated with long-term all-cause mortality in patients at high risk for coronary artery disease with coronary angiography [31]. The advantage of PLR is that it reflects the condition of patients with both inflammatory and thrombosis pathways [32]. It is more valuable than a platelet or lymphocyte count alone. At the same time, PLR is an easy indicator to obtain and easy to follow up.

In this study, multivariate Cox regression analysis showed that smoking history, CVD history, hemoglobin, serum albumin, serum calcium, and serum potassium were the risk factors of PDRP. The results of this study are consistent with previous findings that hypokalemia and hypoalbuminemia are associated with an increased risk of peritonitis. Serum albumin and hemoglobin as nutritional and muscle mass surrogate measures have been confirmed to be related to the occurrence of PDRP [33–35]. Meanwhile, history of smoking and CVD were also mentioned as risk factors for PDRP in some single-center retrospective studies [36, 37].

There are some limitations in our study. First, this is a retrospective study of several provinces in southern China and cannot draw causal conclusions, implying limited generalizability. Second, this study only divided the population into two groups according to the cut-off value, and could not observe whether minimum values of the PLR may be equal to higher PLR for PDRP in PD patients. Third, the database lacked iron-deficiency related parameters and could not rule out the influence of iron-related indexes on platelets. Finally, PLR could not be compared with C-reactive protein, procalcitonin (PCT), IL-6, and other related indicators. The lack of demographic data such as sanitary conditions and education levels in the database may bias the results. Therefore, it is necessary to conduct prospective studies for different populations to determine the best PLR value and make better use of this simple hematological index.

## Conclusion

To sum up, this is a study to examine the relationship between PLR and new-onset PDRP events in PD patients. PLR, as a readily available hematological marker, was associated with peritonitis in patients undergoing peritoneal dialysis.

## Supplementary Information

**Additional file 1.**

**Additional file 2.** 

## Data Availability

The datasets used and/or analyzed during this study are available from the corresponding author on reasonable request.

## Abbreviations

PLR: Platelet-to-lymphocyte ratio
PD: Peritoneal dialysis
PDRP: Peritoneal dialysis-related peritonitis
CAPD: Continuous ambulatory peritoneal dialysis
CIF: Cumulative incidence function
ROC: Receiver operating characteristic
LPG: Low PLR group
HPG: High PLR group
CKD: Chronic kidney disease
ESRD: End-stage renal disease

## References

[1] Jain AK (2012). Global trends in rates of peritoneal dialysis. J Am Soc Nephrol.

[2] Boudville N (2012). Recent peritonitis associates with mortality among patients treated with peritoneal dialysis. J Am Soc Nephrol.

[3] Li PK, et al. ISPD Peritonitis Recommendations: 2016 Update on Prevention and Treatment. Peritoneal dialysis international : journal of the International Society for Peritoneal Dialysis. 2016;36(5):481–508. 10.3747/pdi.2016.00078 PMC5033625 27282851

[4] Cho Y, Johnson DW (2014). Peritoneal dialysis-related peritonitis: towards improving evidence, practices, and outcomes. Am J Kidney Dis.

[5] Perl J (2020). Peritoneal Dialysis-Related Infection Rates and Outcomes: Results From the Peritoneal Dialysis Outcomes and Practice Patterns Study (PDOPPS). Am J Kidney Dis.

[6] Johnson DW (2009). Association of dialysis modality and cardiovascular mortality in incident dialysis patients. Clin J Am Soc Nephrol.

[7] Davies SJ (1998). What really happens to people on long-term peritoneal dialysis?. Kidney Int.

[8] Szeto CC, Li PK (2019). Peritoneal Dialysis-Associated Peritonitis. Clin J Am Soc Nephrol.

[9] Liakopoulos V (2017). Peritoneal dialysis-related infections recommendations: 2016 update. What is new?. Int Urol Nephrol..

[10] Yang X (2018). High Intraperitoneal Interleukin-6 Levels Predict Peritonitis in Peritoneal Dialysis Patients: A Prospective Cohort Study. Am J Nephrol.

[11] Diem S (2017). Neutrophil-to-Lymphocyte ratio (NLR) and Platelet-to-Lymphocyte ratio (PLR) as prognostic markers in patients with non-small cell lung cancer (NSCLC) treated with nivolumab. Lung Cancer.

[12] Wang DS (2012). Comparison of the prognostic values of various inflammation based factors in patients with pancreatic cancer. Med Oncol.

[13] Liu D (2022). The Value of Platelet-to-Lymphocyte Ratio as a Prognostic Marker in Cholangiocarcinoma: A Systematic Review and Meta-Analysis. Cancers..

[14] Zhang T (2020). Risk factors and predictors associated with the severity of COVID-19 in China: a systematic review, meta-analysis, and meta-regression. J Thorac Dis.

[15] Zhao C (2020). Prognostic value of an inflammatory biomarker-based clinical algorithm in septic patients in the emergency department: An observational study. Int Immunopharmacol.

[16] Catabay C (2017). Lymphocyte Cell Ratios and Mortality among Incident Hemodialysis Patients. Am J Nephrol.

[17] Zeng M (2020). J-shaped association of platelet-to-lymphocyte ratio with 5-year mortality among patients with chronic kidney disease in a prospective cohort study. Int Urol Nephrol.

[18] Chen T, Yang M (2020). Platelet-to-lymphocyte ratio is associated with cardiovascular disease in continuous ambulatory peritoneal dialysis patients. Int Immunopharmacol.

[19] Sheng H (2022). Sexual Effect of Platelet-to-Lymphocyte Ratio in Predicting Cardiovascular Mortality of Peritoneal Dialysis Patients. Mediators Inflamm.

[20] Szeto CC (2017). ISPD Catheter-Related Infection Recommendations: 2017 Update. Perit Dial Int.

[21] Classification and Diagnosis of Diabetes (2020). Standards of Medical Care in Diabetes-2020. Diabetes Care.

[22] Jones NR (2020). Diagnosis and management of hypertension in adults: NICE guideline update 2019. Br J Gen Pract.

[23] Li PK, Ng JK, Mcintyre CW (2017). Inflammation and Peritoneal Dialysis. Semin Nephrol.

[24] van der Meijden P, Heemskerk J (2019). Platelet biology and functions: new concepts and clinical perspectives. Nat Rev Cardiol.

[25] Fang T (2020). Diagnostic Sensitivity of NLR and PLR in Early Diagnosis of Gastric Cancer. J Immunol Res.

[26] Rubenstein DA, Yin W (2018). Platelet-Activation Mechanisms and Vascular Remodeling. Compr Physiol.

[27] Koupenova M (2018). Circulating Platelets as Mediators of Immunity, Inflammation, and Thrombosis. Circ Res.

[28] Rossaint J, Margraf A, Zarbock A (2018). Role of Platelets in Leukocyte Recruitment and Resolution of Inflammation. Front Immunol.

[29] Datta S, Sarvetnick N (2009). Lymphocyte proliferation in immune-mediated diseases. Trends Immunol.

[30] Wang J (2018). Prognostic role of platelet to lymphocyte ratio in prostate cancer: A meta-analysis. Medicine (Baltimore).

[31] Lee Y (2018). Usefulness of Platelet-to-Lymphocyte Ratio to Predict Long-Term All-Cause Mortality in Patients at High Risk of Coronary Artery Disease Who Underwent Coronary Angiography. Am J Cardiol.

[32] Balta S, Demirkol S, Kucuk U (2013). The platelet lymphocyte ratio may be useful inflammatory indicator in clinical practice. Hemodial Int.

[33] Zanger R (2010). Hyponatremia and hypokalemia in patients on peritoneal dialysis. Semin Dial.

[34] Davies SJ (2021). Low Serum Potassium Levels and Clinical Outcomes in Peritoneal Dialysis-International Results from PDOPPS. Kidney Int Rep.

[35] Obi Y (2018). Impact of Obesity on Modality Longevity, Residual Kidney Function, Peritonitis, and Survival Among Incident Peritoneal Dialysis Patients. Am J Kidney Dis.

[36] Hu S (2018). Peritonitis: Episode Sequence, Microbiological Variation, Risk Factors and Clinical Outcomes in a North China Peritoneal Dialysis Center. Kidney Blood Press Res..

[37] Karagulle IV (2013). Risk factors for peritonitis related to peritoneal dialysis. Bratisl Lek Listy.

