# Supplementary material for: Platelet-to-lymphocyte ratio and the first occurrence of peritonitis in peritoneal dialysis patients
Source: BMC Nephrol. 2022 Dec 30;23:415. doi: 10.1186/s12882-022-03038-5 (PMC9803258; doi:10.1186/s12882-022-03038-5)
Supplement: Supplementary file 1 — Additional file 1. [file 12882_2022_3038_MOESM1_ESM.docx]

|  | | | | |
| --- | --- | --- | --- | --- |
|  | Death | Transform to HD | Renal transplantation | P-value |
| Numbers | 284 | 157 | 72 |  |
| **Demographics** |  |  |  |  |
| Age(years) | 57.42±14.03 | 50.31±14.93 | 38.65±11.09 | <0.001 |
| Male（%） | 157(55.3%) | 106(67.5%) | 49(68.1%) | 0.017 |
| Smoke (%) | 3(1.1%) | 4(2.5%) | 3(4.2%) | 0.189 |
| BMI (kg/㎡) | 22.02±3.46 | 22.85±3.33 | 21.64±3.03 | 0.056 |
| **Comorbidities** |  |  |  |  |
| Hypertension (%) | 228(80.3%) | 118(75.2%) | 48(66.7%) | 0.042 |
| Diabetes (%) | 113(39.8%) | 37(23.6%) | 7(9.7%) | <0.001 |
| History of hyperlipemia (%) | 24(8.5%) | 7(4.5%) | 2(2.8%) | 0.104 |
| History of CVD (%) | 39(13.7%) | 12(7.6%) | 1(1.4%) | 0.004 |
| **Laboratory Variables** |  |  |  |  |
| Total Kt/V | 2.09(1.58-2.59) | 2.04(1.41-2.74) | 2.25(1.58-2.72) | 0.150 |
| Albumin (g/L) | 34.85(30.60-38.30) | 35.10(32.30-35.10) | 35.25(32.10-39.55) | 0.006 |
| RRF（mL/min/1.73㎡） | 3.32(1.76-5.60) | 3.37(1.82-5.56) | 2.77(1.61-5.24) | 0.176 |
| WBC (×10^9^/L) | 5.65(4.30-7.79) | 5.96(4.90-7.60） | 6.30(4.53-7.32） | 0.280 |
| RBC (×10^12^/L) | 2.79(2.40-3.19) | 2.65(2.37-3.01） | 2.92(2.39-3.32 | 0.004 |
| Hemoglobin(g/L) | 80.00(72.00-91.00) | 77.00(68.25-87.00） | 83.00 (69.75-94.50) | 0.001 |
| Lymphocyte (×10^9^/L) | 1.08(0.82-1.46） | 1.17(0.85-1.57） | 1.23(1.03-1.41) | 0.476 |
| Neutrophil (×10^9^/L) | 3.78(2.76-5.40） | 4.40(3.23-5.14) | 4.12(2.98-5.42) | 0.101 |
| Platelet (×10^9^/L) | 160.00(110.75-230.25） | 168.50(133.00-207.00) | 170.50(112.75-180.97) | 0.624 |
| PLR | 143.49(101.89-196.16) | 148.34(103.60-213.93) | 139.14(94.20-196.79) | 0.564 |
| FBG (mmol/L) | 4.70(4.10-5.90） | 4.42(3.99-5.40) | 4.71(4.32-5.63) | <0.001 |
| Urea nitrogen(mmol/L) | 19.75(15.13-25.90） | 22.69 (15.83-28.98) | 21.33(14.85-27.58) | 0.007 |
| Calcium (mmol/L) | 2.01(1.82-2.14) | 1.95(1.76-2.07) | 2.04(1.86-2.18) | <0.001 |
| Phosphorus (mmol/L) | 1.60(1.34-1.95) | 1.85(1.56-2.08) | 1.82(1.37-2.09) | 0.001 |
| iPTH (pg/ml) | 156.42(92.08-275.47) | 188.73(119.99-272.00) | 229.29(113.52-360.18) | 0.449 |
| Total cholesterol（mmol/L ） | 4.20（3.26-5.10） | 4.21(3.21-4.95) | 4.10(3.44-4.88) | 0.708 |
| Triacylglycerol(mmol/L) | 1.34（0.88-1.75） | 1.27(0.86-1.64） | 1.32(0.96-2.09) | 0.761 |
