# Supplementary material for: Platelet-to-lymphocyte ratio and the first occurrence of peritonitis in peritoneal dialysis patients
Source: BMC Nephrol. 2022 Dec 30;23:415. doi: 10.1186/s12882-022-03038-5 (PMC9803258; doi:10.1186/s12882-022-03038-5)
Supplement: Supplementary file 2 — Additional file 2. [file 12882_2022_3038_MOESM2_ESM.docx]

| Attachment 2. Table for the use of iron and erythropoietin in the two groups. | | | |
| --- | --- | --- | --- |
|  | PLR≥161.5 | PLR<161.5 | P-value |
| EPO | 414(76.8%) | 556(78.1%) | 0.591 |
| Iron agent | 381(70.7%) | 499(70.1%) | 0.817 |
